# Optical modelling of a supplementary tunable air-spaced goggle lens for rodent eye imaging

**DOI:** 10.1371/journal.pone.0181111

**Published:** 2017-07-20

**Authors:** Elie de Lestrange-Anginieur, Xiaoyun Jiang, Qiushi Ren

**Affiliations:** Department of Biomedical Engineering, College of Engineering, Peking University, Beijing, P.R. China; University of Rochester Medical Center, UNITED STATES

## Abstract

Aberration variations severely degrade retinal imaging in small animal eyes. Previously, the approach of a goggle lens with a matching corneal index was proposed to overcome the on-axis resolution limit of static imaging systems, which allows the use of the full eye pupil. But this technique didn’t address the problem of the large power variation, and the ensuing aberration on and off-axis, when dealing with small animal eyes. In this study, we present the concept of a tunable goggle lens, designed to compensate individual ocular aberration for different rodent eye powers. Ray tracing evidences that lens-fitted goggles permit, not only to adjust individual eyes power, but also to surpass conventional adaptive correction technique over large viewing angle, provided a minimum use of two spaced liquids. We believe that the overlooked advantage of the 3D lens function is a seminal finding for further technological advancements in widefield retinal imaging.

## 1 Introduction

Imaging of the rodent eye has received particular attention in the context of fundus disease. Owing to a numerical aperture more than two times that of the human eye, rodent eyes offer higher spatial resolution [1]. However, in practice, the high optical power of the eye, and its ensuing high amount of spherical aberration and coma [2], inevitably degrade the retinal image quality, which precludes the use of the full pupil of the eye with a standard lens system [3]. To draw the optimal resolution promised in small animal eyes, the incorporation of a wavefront corrector (e.g., deformable mirror) using a conjugate adaptive optics (AO) system becomes essential. The observation of photoreceptoral cells in vivo [4] is yet restricted to a very narrow field angle, and can rapidly involve very complex system architecture when correcting multiple fields, outside the isoplanatic patch of the eye [5]. A competitive and somewhat complementary alternative to external correction is provided by contact correction. To minimize aberrations, a direct compensation can be achieved at the site of the eye using a contact element such as a rigid contact lens [6]. Nonetheless, the selection of an optimal optical design and its potential benefit seems to have received little attention in the field of retinal imaging. In fact, the concept of an ‘artificial cornea’ in retinal imaging was propounded only tardily with the proposal of a corneal matching index goggle lens [7]. By reducing corneal aberration, the authors demonstrate that the immersion of the cornea in a suitable liquid (i.e. saline water) could allow a substantial improvement in retinal images, resulting in a larger optical throughput with increased resolution on axis. An impediment to the extension of this approach was caused by the additive components required within the system (e.g. aspheric plate), so a fine-tuned compensation of individual aberrations, and its extension for off-axis retinal images remained concealed.

For imaging rodent eyes, the incorporation of an optical probe element having a variable power in front of the cornea [8], was proposed by our group, to account for the large power variation (refractive errors range: +5 to +15D [9]), found in rodent eyes, as compared to the human eye. The realization of a separate corrective unit in the contact probe brought a strong stabilization of the eye [8, 10]. Moreover, it afforded to spatially extend the artificial cornea beyond the restrictive physical limit of a lens-fitted contact lens (i.e. having ordinarily lighter weight). That creates a opening for fine tune control of the optical aberrations of the rodent eye. Given that the use of solid glass restricts an optimal correction across rodent eyes, we were interested in the imaging performance allowed by an adaptive corneal compensation. So far, however, there is no published design for a flexible artificial cornea, capable ofcompensating different rodent eyes, nor is there an indication of the potential of this type of contact correction, if any, with respect to an indirect correction (i.e. within the system itself).

To answer the question of a tunable artificial cornea, a special category of adaptive optics system can be considered: the optofluidic lenses [11]. In deformable membrane lenses, an optical liquid concealed in a distensible membrane undergoes a change of pressure that causes the membrane to curve, hence permitting a continuous transformation of the wavefront of the light over space. The transformation of the wavefront relies on the material composition (e.g. liquids coated with thin elastomeric membranes), the lens geometry (e.g. spherical), as well as actuation mechanism (e.g. fluidic pressure, pneumatic actuation, mechanical stress), which is currently the object of considerable research [12]. The optofluidic lens technology demonstrates a significant potential, not only for defocus correction, but many other aberrations such as astigmatism [13], which makes it a highly promising tool for clinical and ophthalmic applications today, in particular for artificial ocular lenses [14–15].

In this study, we demonstrate that the use of an air-space liquid lens, serving as an artificial cornea, could present a significant potential to enhance wide-angle retinal images (≈20deg) of the rodent eye, and complement indirect imaging techniques. The concept is demonstrated using optical modeling software, not demonstrating a real (physical) lens system, therefore there may well be manufacturing considerations that need to be taken into account before instantiation can be realized.

## 2 Methods

**Fig 1:**
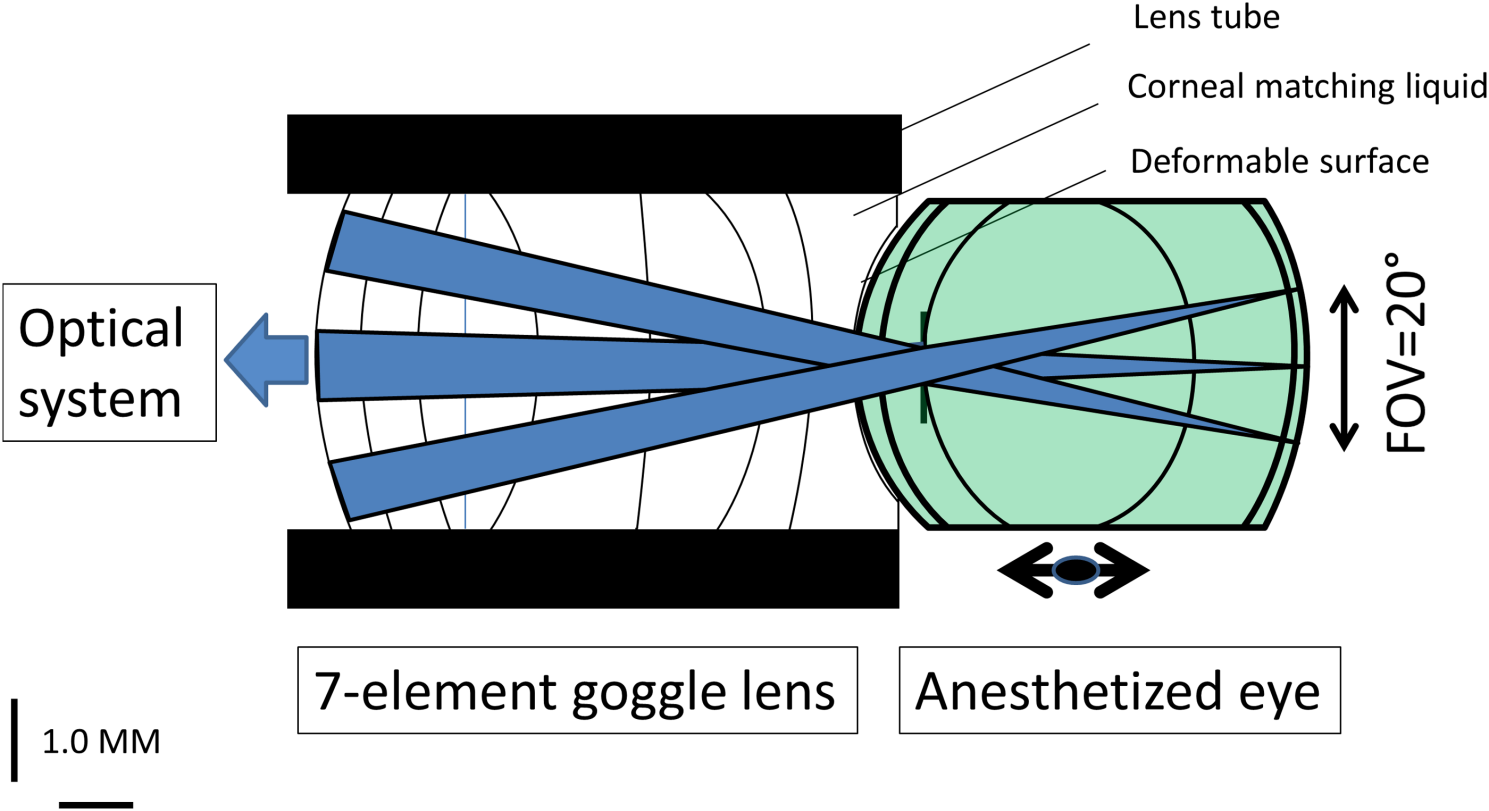
Example of a multi-element lens-fitted goggle rigidly fastened to an optical system: The goggle lens is made of a plurality of liquid filled cavities, having a distinct refractive index, separated by elastic surface membranes that enable a static correction of the eye by restructuration of the rodent cornea. The rodent cornea is placed in soft contact with the back surface of the goggle lens, having a corneal matching index (see [8] for details) meant to minimize the corneal wavefront errors. An assessment of a direct correction method is provided by the number of surfaces necessary to correct the eye, which gives an indication of the practicability of a tunable goggle lens design.

The optical design software ZEMAX version 2009 (Zemax Development Corporation) is used to investigate an optimum lens-fitted goggle design, and its capability for imaging the rodent eye. We use a rodent schematic eye developed by Zhou et al. [16] based on radius of curvature and gradient index lens (GRIN) data obtained from Campbell and Hughes (1981) [17], and surface asphericity data from Chaudhuri et al. (1983) [18]. This model is chosen for its ability to account for the impact on aberration of surface asphericity, gradient index lens, and high numerical aperture. The wavelength for imaging is set to 550nm. Several entrance pupils are tested to account for the gradation of aberration and its determining impact on the retinal image quality. Because of the dominant aberration contribution of the highly powered eye, the imaging system is regarded as diffraction limited and modeled by a simple paraxial lens. The emmetropic eye -corrected externally in the system, is taken as a reference eye. In the indirect correction, light exiting the objective lens of the system is immediately focused onto the myopic eye whereas in the contact correction, light exiting the objective lens is first collimated onto the goggle lens, which then compensate for the eye defocus. The goggle lens, similar to a contact lens, is placed in soft contact with the anterior cornea where it acts as an artificial cornea that restructures the eye aberration. An illustration of the optical layout is depicted in Fig 1 for a multi-element goggle lens design.

By fastening the goggle lens onto the imaging system [8] next to the exit pupil of the system, only the eyeball needs to be adjusted along the optical axis of the system; this was previously shown to increase stability and fixation of the rodent eye [8, 10]: hence, a maximum intensity recording can be considered onto the detector under anesthesia of the rodent eye. The attachment of the goggle lens permits the placement of an artificial eye with a more advanced optomechanical design as compared to a lens-fitted contact lens, but might cause some differences in intraocular pressure [19–20], which could affect the integrity of structural elements within the retina as well as some functions (e.g. change blood flow) over long-term time course imaging. Two lens-fitted corrections are investigated and compared to the reference eye: an index matching-based and refractive index mismatch optical correction. In the former strategy, the cornea is immersed in a liquid element, having identical refractive index and complemented by a power lens assembly compensating for the large refractive error of the myopic rodent eye. The performance of the ‘goggle lens wearer eye’ is determined by the number of surfaces element of the ‘power lens group’, the thicknesses, the curvatures and the refractive indices within each fluidic cavity. Each liquid-filled cavity is enclosed between two independently varying surfaces, and the deformation of the optical surface is simulated by a simple change of curvature.

**Fig 2:**
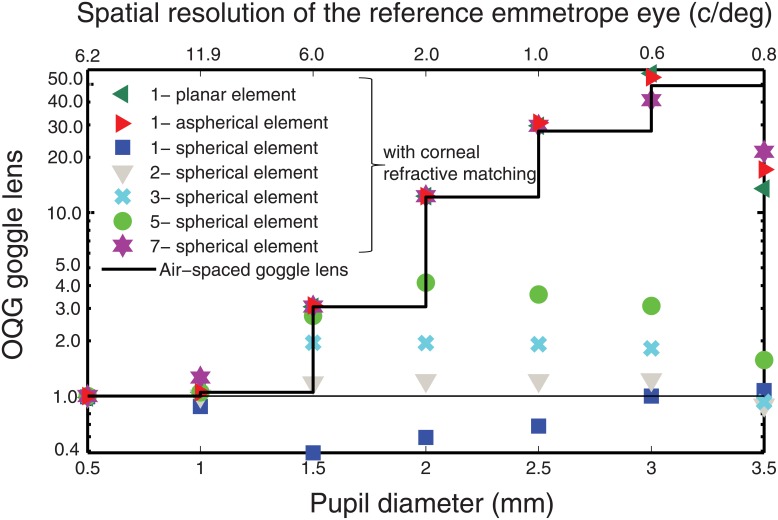
Optimum performance allowable by a goggle lens correction: On-axis optical quality gain OQG of various liquid lenses via corneal index matching optical correction. The stairs function corresponds to the alternative solution of a ‘non-corneal matching index’ with a 2-element air-spaced goggle lens. The performance by indirect optical correction at various pupil diameters appears on the upper axis.

To maintain the generality of our analysis, the geometry of the compartment was deliberately neglected. For each k-element goggle lens, a discrete set of refractive index values was elected, which ranges between 1.33 to 1.67, as typical of a transparent liquid [21]. Various copies of the k-element goggle lens are constructed by permutation of those refractive index values. For each copy of a k-element goggle lens, trial and errors were then used to determine the optimum curvature and position of the refractive surfaces. The comparison of the elected k-element goggle lens yields an evaluation of the minimum number of deformations required for an ideal correction. Fig 2 shows the optical quality gain (OQG) achievable by each goggle lens, which is quantified as the ratio of direct to indirect optical quality of the correction (OQ):

$$OQG=\frac{OQ_{direct\;compensation}}{OQ_{indirect\;compensation}}$$

In the indirect correction, only the spherical defocus component of the rodent eye is corrected (e.g., by an objective lens), hence the eye is emmetropic. In the direct correction, the goggle lens however compensates for defocus and other aberrations by altering the light focusing structure of the front of the eye. The optical quality (OQ) of the correction is measured in image space by using the Fast Fourier Transform modulation transfer function FFT MTF. In order to relate the optical quality to imaging of the cellular structures in the retina, the system performance is reported using the optimal ocular resolution of the rodent eye, as measured by the maximum spatial frequency falling at 0.5 MTF (Figs 2 and 4–6). The spatial resolution is optimized by evaluating the RMS spot radius merit function, followed by the RMS wavefront merit function. The outer limiting membrane of the retina is selected as the image plane, which implies a preferential depth of interest within the thick retina. It is important to note that it is a simplification because, in practice, an optical correction at a given retinal depth will be impacted by the degree of matching between the sensing plane (used for correction) and the actual imaging plane [16].

**Fig 3:**
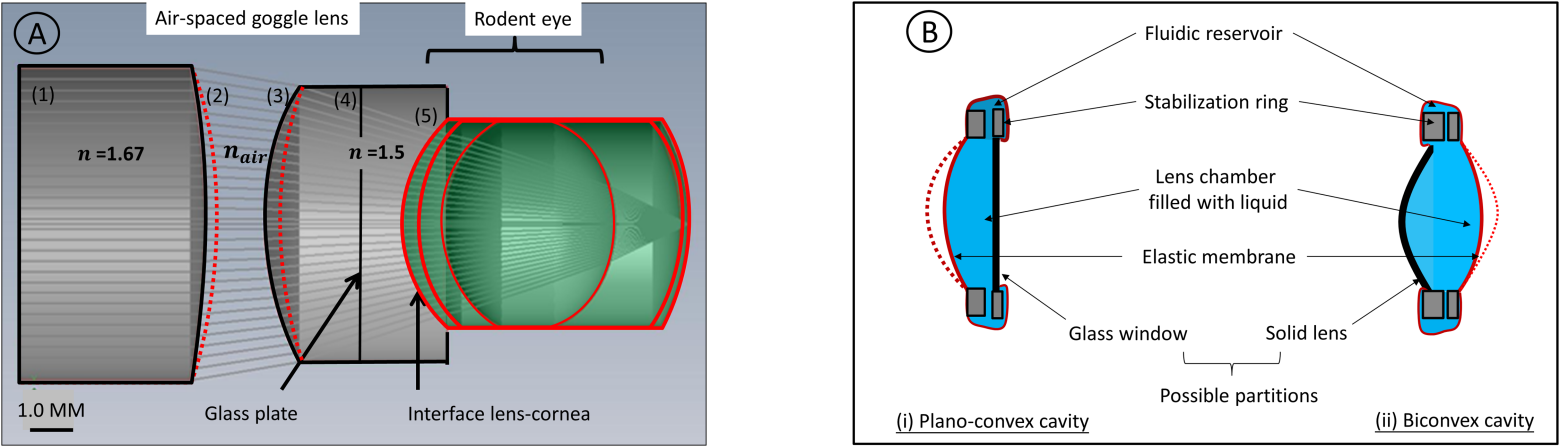
Plano air-spaced liquid goggle lens for application in retinal imaging: A/ The plano air-spaced liquid goggle lens consists of two refractive index n1 and n2, split into different optical cavities. At the back of the goggle lens, the first cavity (4–5) introduces a compensatory refractive index mismatch, while on-axis and off-axis residual aberrations are corrected by an auxiliary unit comprising two optical cavities (1–2) and (3–4), with two surface deformations (2) and (3). B/ Each cavity is enclosed between an elastic membrane and a solid partition via a ring holder that allows a push/pull movement of the liquid from the cavity to the reservoir. Thanks to the limited number of elements, the solid partition allows the individual actuation of the surface by fluidic pressure. Different configurations are possible for the front cavity (1–2): (i) for a plano air-spaced goggle lens, the partition is simply a glass plate, (ii) for a convex air-spaced liquid goggle lens, a solid lens is placed at the front of the liquid lens as a replacement of the front glass plate.

To further evaluate this method, we examined the alternative of a refractive index mismatch at the lens-cornea transition by immersion of the cornea in a media with high refractive index (n = 1.5). The mismatch-induced aberration cancels out the major contribution of the wavefront deformation in the eye (i.e. about 46% of the total on-axis wavefront at the largest pupil). This primary compartment is enclosed between an elastic membrane (abutting the cornea) and a glass window via a stabilizing ring, as shown in Fig 3. The membrane bends in response to the corneal contact (i.e., push or pull), forcing the liquid to enter or exit the cavity by communication with a reservoir, formed by the ring holder. The glass window separates this compartment from an auxiliary unit situated in the front of the lens, which further compensates the residual aberration. This unit comprises a total of two deformable curvatures separated by an air-space, and ended by a solid front window, planar or curved. All in all, the air-spaced goggle lens is made of two refractive index liquids segregated into individual chambers having a solid-liquid structure, which permits the independent deformation of the surface.

**Fig 4:**
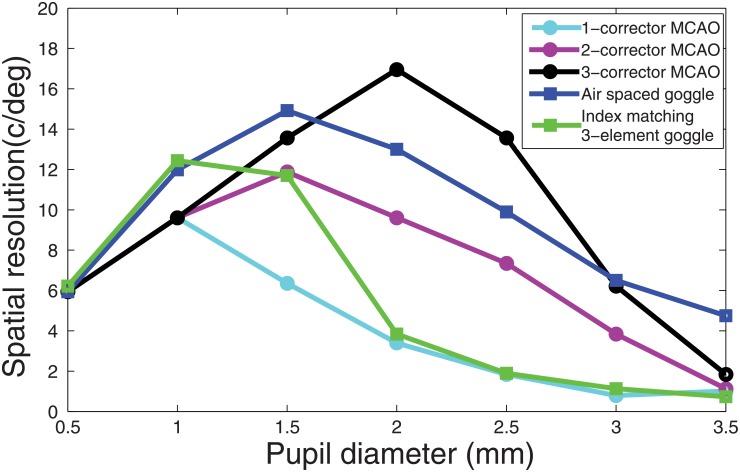
Comparison of the performance of various optical correctors: Wide-angle resolution achieved, over a +/-10 degree field angle, as a function of pupil diameter, using a planar, distant phase conjugation approach, for a varying number of n-corrector MCAO [23], and the alternative of a contact correction by a tunable goggle lens. The restructuration of the rodent eye by a mismatched index method exhibits competitive performance compared with phase conjugated methods using thin phase plates correctors.

The lens solution is compared with current adaptive correction, based on phase-conjugation. The action of an active matrix element (e.g. deformable mirror) is simulated by a thin phase plate conjugated to the anterior corneal surface [22], and described by 20 Zernike modes using the “Zernike standard phase” surface in Zemax. In order to fully appreciate the potential of the lens solution alternatives, a multi-conjugate adaptive optics (MCAO) [23] correction is tested by sequentially incrementing the number of mirror conjugations across the refractive bulk of the eye. In the 2 MCAO, the former single corrector is supplemented by a second mirror positioned near the retina (i.e., to a point 4.59mm inside the eye). For the 3 MCAO, the 2 set mirrors are further complemented by a third mirror placed at an intermediate position between the cornea and the retina (i.e., to a point 0.88mm inside the eye). Each extra corrector was optimized in turn, thus assessing the relative gain of an added corrector. The FFT MTF was calculated for four distinct field heights, at 0 mm, 0.21mm, 0.43 mm, and 0.64 mm corresponding to field angles of 0°, 3.33°, and 6.6°, and 10° respectively. For the wide-angle retinal image, each field was given equal weight in our merit function. The optical quality (OQ) of the correction is measured as the wide-angle resolution, quantified as the lowest resolution in the field at 0.5 MTF. Fig 4 compares the wide-angle resolution of the correction of the MCAO and the goggle lens alternative as a function of pupil size. The number of mirrors necessary affords an insightful comparison with the number of surfaces used in the goggle lens design. Note however that the placement of the mirror was somewhat arbitrary, and other axial distributions may be set according to the geometrical requirements of the system.

## 3 Degree of freedom required by a goggle lens correction with a corneal matching index

If an indirect correction can compensate spherical defocus, it often fails to reduce other ocular aberrations. Although conjugate adaptive systems can considerably enhance the resolution of wide-angle images, the amount of light detection might be sacrificed because of the insertion of extra optical relays in the system. A reduction of complexity could be envisaged by the use of contact lenses. Contact lens is common routine in retinal imaging where it helps to maintain corneal hydration and can assist an indirect correction by reducing defocus. However, the optimal quality attainable using a goggle lens, as well as the number of surfaces needed is still uncertain. Therefore, it would be interesting to know whether an advanced goggle lens could correct the aberration of the rodent eye, and if yes what design alternative could permit the transition to that tunable goggle lens.

A pioneer study showed that, by designing an artificial goggles lens with a corneal index matching, a significant improvement for on-axis resolution images is achievable for the human eye. Fig 2 confirms that corneal index matching [7] tremendously improves on-axis resolution for the rodent eye, making a plano goggle lens an ideal candidate for reducing corneal optical errors in the emmetropized rodent eye (i.e. external correction). Immersion of the cornea is however insufficient when a direct defocus correction is applied on a single lens element (e.g. contact lens), and requires aspherizing the lens surface. Considering the technical difficulty of tuning surface asphericity, the addition of refractive elements could present a potential alternative for aberration control. A versatile artificial cornea however seems to yield poor benefit in terms of aberration correction. Via a 2 and 3-elements index-matching-based goggle lens, a modest improvement in image quality is shown as compared to an indirect optical correction (see Fig 2, OQG<2). With further elements, the gain of an artificial cornea is significantly augmented. For a 5 element-goggle lens, a 4 fold increase in spatial resolution is anticipated with respect to indirect correction for a 2mm diameter pupil, yielding a resolution of about 4x2.0 = 8cycle/deg. This is however still smaller than the optimum performance of 2.7x6.0 = 16.4cycle/deg achieved when narrowing pupil diameter to 1.5mm. As a matter of fact, the advantage of a larger pupil area is only restituted via the 7-element-goggle lens with a spatial resolution of 25.4cycle/deg for a 3mm diameter pupil. Nonetheless, the large number of lens elements restricts a versatile design. In order to simplify the implementation of an adaptive goggle lens, a new approach is necessary.

## 4 Mismatched index approach

An alternative solution is proposed using a refractive-index-mismatch induced aberration compensation. It uses an air-spaced goggle lens. A refractive index difference between the anterior cornea and the back window of the lens-fitted goggle (Fig 3, Table 1) is purposely introduced to restructure the aberration of the eye. A strategic air-space permits the free deformation of 2 auxiliary surfaces, which compensate the modified ocular wavefront. With its 2 liquid elements (Fig 3, Table 1), the air-space goggle lens yields far improved performance compared with index matching-based optical corrections on-axis [7] (Fig 2), theoretically allowing the use of the full pupil of the eye. (Table 1).

**Table 1:** Specification of a plano air-spaced liquid goggle lens.

Lens type	Glass plate	Cavity (1–2)		Air space	Cavity(3–4)	Glass plate	Cavity (4–5)	
**Surface no**	**1**		**2**		**3**	**4**		**5**
**Radius (mm)**	**inf**		**14.277**		**5.099**	**inf**		**R cornea**
**Thickness(mm)**	**0**	**4**		**1.287**		**0**	**3**	
**Refractive index**		**1.67**		**1**			**1.5**	

This improvement is further established in wide-angle condition, where an improved spatial resolution is found across pupil conditions, using a convex air-spaced goggle lens. A gain in resolution up to 5 times with respect to the index-matching-based 3-element goggle lens is anticipated at an intermediate pupil diameter of 2mm (Figs 2 and 4). Furthermore, an adaptive optics correction is lagging behind a direct optical correction of wide-angle images as shown by the flat performance of a single mirror correction across pupil size. Indeed, multiple correction adaptive optics gradually improved a conjugate adaptive correction, with an increase in performance particularly appreciable when pupil are larger than 2mm. The advantage of a basic artificial cornea suggests that the liquid lenses may provide a complement for multiple spaced conjugate wavefront correctors when correcting more than one aberration.

**Fig 5:**
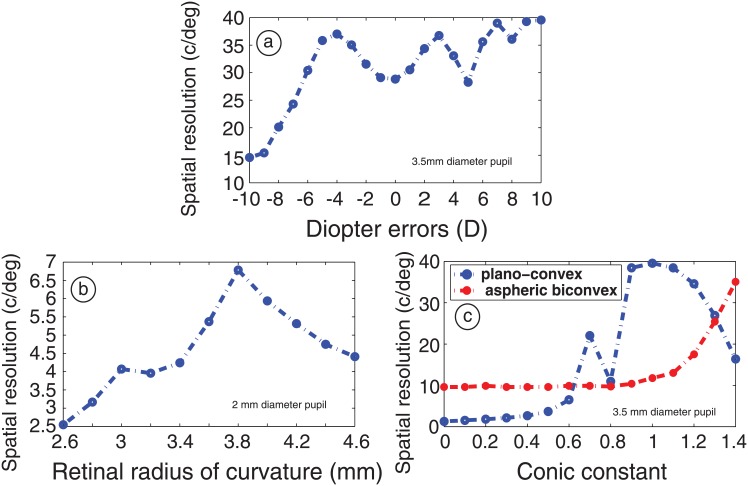
Tuning range of an air-spaced goggle lens: On-axis optical resolution achieved by a/ a plano air-spaced goggle lens correction as a function of simulated refractive errors variations (i.e. in axial length) and b/ retinal curvature. c/ On-axis optical resolution of a plano and a convex aspherical goggle lens correction, as a function of corneal asphericity of the rodent eye.

Knowing that the two-air spaced lenses allow high resolution imaging on and off-axis, an important concern is whether an adaptive goggle lens remains conditional to the description of the eye architecture, or can adjust to the large structural variations in the rodent eye. To elucidate this point, several geometrical transformations are added into the initial model eye, which simulate changes of the ocular geometry (Fig 5) [17]. The data shows that an air-space goggle lens is able to maintain a stable compensation across large power variations, even at the larger pupil of 3.5mm. At larger retinal eccentricities, a change in retinal curvature exhibits a strong impact on performance, indicating that structural changes may affect the optical prediction. In addition, when the full pupil of the rodent eye is used, the corneal asphericity of the rodent eye becomes critical: an air-space goggle lens having a planar front surface exhibits an optimal optical performance for oblate corneal shapes (i.e., conic value Q = 1), but very poor performance for the spherical corneal shape, as shown in Fig 5c. By replacing the front window of the air-spaced goggle lens by an aspherical convex surface (conic value Q = -10.5, radius of curvature R = 18mm), the optical performance is maintained over a broad range of corneal asphericities, which attenuates a reduction in performance at the largest pupil. It results that a liquid-based goggle lens joint to one aspherized rigid surface might adapt the basic geometrical variations of various rodent eyes to permit bigger imaging pupil.

## 5 A supplementary goggle lens for enhanced wide-angle resolution

**Fig 6:**
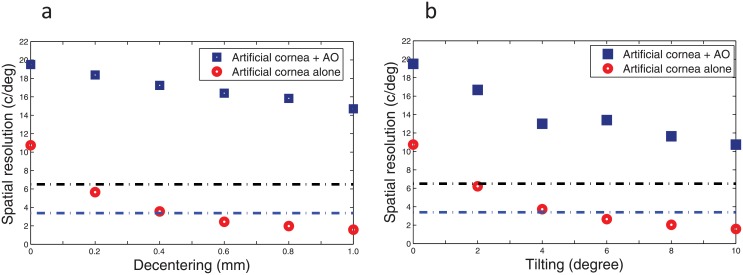
Robustness to lens fitting errors: Wide-angle resolution achieved for a 2-mm pupil diameter over a +/-10 degree field angles using a tunable artificial cornea alone (red symbols) and in combination with a conjugated adaptive plano-element (blue symbols) conjugated to the pupil of the eye, as a function of a) decentering and b) tilting of the goggle lens with respect to the eye pupil. The black and blue dashed baselines indicate the performance achieved using a single AO corrector conjugated to the eye pupil and the cornea, respectively. Below those 2 baselines, the performance of an adaptive correction using a single plano-element dominates an artificial cornea correction. Note that association of AO with an artificial cornea brings an outstanding performance benefit for wide-angle images, as compared to an artificial cornea alone and a single AO corrector alone.

Indeed, the optical strategy of an artificial cornea, to be effective, must rely on a good control of the alignment of the eyeball on the goggle lens, in analogy to aberration pattern generation in AO visual simulation [24]. Hence, an excellent centration is required for an artificial cornea correction. This can be best achieved under Lab experiment when the goggle lens is fastened onto the system and lens alignment monitored by moving the eye with respect to the goggle lens fixed [8,10]. In fact, for an anesthesized eye, blink reflexes can be appreciably diminished, and even abolished [25], which permits an optimal and steady placement of the lens for the use of an artificial cornea. However, in absence of sufficient level of anesthesia, blinks are expected to affect the optimal optical performance of an artificial lens alone, as shown in Fig 5. With blinking, typical decentration, although often considerably less than 0.5mm (for contact lenses wearer [26]), could reduce an ideal contact correction performance by a factor of more than 3 for a 1.0mm decentration, thus reducing the optical advantage of an artificial cornea over a single AO corrector in Fig 5. For stronger misalignment errors (e.g., >0.5mm), an artificial cornea to hold the promise of an enhanced field of view, may be assisted by the use of an additional adaptive element conjugated to the eye pupil [27]. Fig 6 shows that this unordinary combination can considerably reduce the impact of misalignment errors, and even, enhance the potential of an artificial cornea, as compared to a conjugate adaptive element alone or an artificial cornea alone in wide-angle images. It is conclusive that an adaptive artificial cornea may significantly contribute to enhanced wide-angle resolution when using a conjugate corrector.

## 6 Discussion & conclusion

The rodent eye aberrations limit the imaging resolution achievable at large pupil, restricting the finest features observable with current imaging systems. This study assessed the on-axis and wide-angle optical performance of a contact correction imaged via a diffraction limited system. Our result confirms the potential of refractive index matching in the rodent eye, showing that the use of a multilayer artificial cornea can assist a higher resolution correction. It is therefore expected that imaging of the cellular structures with a designed goggle lens could be a significant improvement over imaging of the uncorrected rodent eyes at large pupil. Indeed, the structural complexity of an opto-mechanical design can be an obstacle to the realization of a tunable goggle lens. When using a refractive index mismatch optical correction, a significant reduction of the number of lens elements is found, which favors the realization of a tunable goggle lens design. We have extended our simulation to show that a goggle lens not only acts on the on-axis aberration, but can also compensates off-axis aberrations by reshaping the optical structure of the rodent eye, which is decisive to broaden the area of enhanced resolution. For the use of a fully dilated pupil, an important limitation remains the existence of additional aberrations that may arise from the individual eye, such as coma or astigmatism, which are not accounted in our model and will limit a theoretical benefit. One practical association of our concept is conjugate adaptive optics, which has revealed a highly efficient technique for imaging cellular and subcellular ganglion cell structures, such as dendritic stratification. Conjugate adaptive optics features a principal advantage (over an adaptive goggle lens) that it can correct higher-order aberrations at a specific retinal field for achieving diffraction limit. By combining the two techniques, we anticipate that the fundamental tradeoff between resolution and broadening of the area of imaging may be ameliorated for accessing a wider retinal view of dynamic physiological processes.

Taken together, by directly restructuring the global balance of the eyeball aberration, instead of externally compensating aberration, we show that a tunable artificial cornea may offer a unique mode of correction. A tunable air-space goggle lens, comprising 2 different liquid elements, exhibits the capability to increase spatial resolution, with a significant expansion of the field typically limited to only few degrees with a planar AO correction [22]. Although the use of a liquid lens in adaptive correction may not have seemed particularly feasible few years ago, with the successful diversification and application of liquid lens in miniature systems (such as intraocular lens [14]), and the parallel development of microsystems engineering, the liquid lens may be the upcoming revolution in the way of compensating optical aberration [12], in particular for wide-angle retinal images. Our result demonstrates via optical modelling that a tunable artificial eye has the potential to correct many aberrations and adjust individual variation in the rodent eye. Supposing that the effect arising from the thick retina, intraocular pressure as well as manufacturing constraints can be overcome, this new concept may open new opportunities for the advancement in wide-angle of high resolution adaptive retinal imaging.
